# “Therapy without a therapist?” The experiences of adolescents and their parents of online behavioural activation for depression with and without therapist support

**DOI:** 10.1007/s00787-023-02142-7

**Published:** 2023-01-17

**Authors:** Rebecca Andersson, Sarah Vigerland, Johan Ahlen, Hanna Widström, Irma Unger, Eva Serlachius, Hedvig Engberg

**Affiliations:** 1 Centre for Psychiatry Research, Department of Clinical Neuroscience, Karolinska Institutet, & Stockholm Healthcare Services, Region Stockholm, Gävlegatan 22, 113 30 Stockholm, Sweden; 2 The Centre for Epidemiology and Community Medicine, Region Stockholm, Box 45436, 104 31 Stockholm, Sweden; 3 Department of Global Public Health, Karolinska Institutet, 171 77 Stockholm, Sweden; 4 Moment Psychology, Drottninggatan 99, 113 60 Stockholm, Sweden; 5 Wemind Psychiatry, Rehnsgatan 20, 113 57 Stockholm, Sweden; 6 Department of Clinical Neuroscience, Karolinska Institutet, 171 77 Stockholm, Sweden; 7 Department of Clinical Sciences, Faculty of Medicine, Lund University, Baravägen 1, 222 40 Lund, Sweden; 8 Department of Women’s and Children’s Health, Karolinska Institutet, Stockholm, Sweden; 9 Department of Obstetrics and Gynaecology, Karolinska University Hospital, Stockholm, Sweden

**Keywords:** Depression, Adolescents, Behavioural activation, Online, Qualitative, Therapist support

## Abstract

Behavioural Activation (BA) is an established treatment for adults with depression, and research on BA for adolescents is promising. However, there is a knowledge gap in terms of the experiences of adolescents and their parents BA for depression delivered online. Furthermore, there have been no previous studies conducted on the experiences of respondents with regard to the role of the therapist in online treatment. Therefore, the primary aim of this study is to explore the experiences of online BA among adolescents with depression and how their parents experience supporting their adolescent through treatment. Second, the experiences of having online therapy with or without a therapist were explored. Semi-structured interviews were conducted with eight adolescents and nine parents (*n* = 17) who completed guided or self-guided online BA. Reflexive thematic analysis was used to identify aspects of the experience of treatment that were important to adolescents and their parents. Two main themes were generated: (1) opportunities or barriers to engaging in treatment and (2) parental involvement is valued and welcomed. This study contributes valuable information regarding user experiences of BA treatment, the importance of therapist support and parental involvement in treating adolescents with depression.

*Trial registration number*: ClinicalTrials.gov Identifier NCT04117789, Date of registration: 07 October 2019.

**Supplementary Information:**

The online version contains supplementary material available at 10.1007/s00787-023-02142-7.

## Introduction

Behavioural activation (BA) is a form of cognitive behavioural therapy (CBT) designed specifically to target depression through an exclusive focus on behaviour change [1]. The BA model suggests that stressful life events, such as experiencing a loss or changing school, can lead to less positive environmental reinforcement [2]. When activities that used to be enjoyable no longer give pleasure, it is natural to cope through avoidance, i.e. withdrawing or cancelling activities. While avoidance offers relief in the short term, it maintains depression in the long term. The treatment begins with self-monitoring, which is an in-depth exploration of the activities that are associated with either feeling positive or feeling down. This is done for the purpose of determining individual targets for the intervention. The client with depression is then encouraged to do more things that will increase contact with activities associated with feeling positive. The patient will be encouraged to get out of bed, leave home, go to school and interact socially even when feeling down, self-critical, empty or hopeless. Through this clients learn to understand the connection between what they do and how they feel. Avoidant behaviour is addressed through engagement in alternative, more adaptive, coping behaviour. Another important part of the treatment is attempting to solve existing life problems, such as being bullied or not having any friends. Towards the end of the therapy, efforts are made to prevent future relapse by identifying early signs of depression, possible pitfalls and defining helpful alternative behaviours.

Unlike traditional CBT, the BA model focuses on a few fundamental behavioural strategies and does not involve interventions aimed at changing cognition. A large body of research shows that BA is an effective and acceptable treatment for adults [1] [3] and due to its simplicity and specific behavioural focus, BA is suggested as being especially well suited to adolescents with depression [4]. Adolescents who have undergone face-to-face BA find treatment rewarding and report increased joy, enthusiasm and motivation [5].

Although adolescent depression is a prevalent disorder [6], only a fraction of young people affected utilise mental health services and even fewer receive psychological treatment [7, 8]. One possible way in which to make psychological treatment more accessible is via online delivery. Previous research has shown that internet-delivered cognitive behavioural therapy (ICBT) is clinically effective for adults with depression [9] and there is growing support for the efficacy of ICBT in children and adolescents with psychiatric disorders [10]. However, it is still unclear if ICBT is an efficient intervention for adolescent depression and there have been no studies conducted investigating online BA for adolescents with depression [11]. Previous qualitative research shows that the experiences of ICBT among young people are mostly positive and that treatment is seen as helpful and flexible [12]. Perceived obstacles to ICBT include content that is too generalised and requires good literacy skills. Patients experience both advantages and disadvantages with the reduced therapist contact that is typical of online CBT [12, 13]. While some patients express feeling alone without a face-to-face therapist, others appreciate working individually with only limited support. Some individuals view ICBT as less stigmatising than talking face-to-face with a professional [12, 14].

ICBT can be delivered as an entirely self-managed intervention or in conjunction with remote therapist support. In a recent meta-analysis comprising 9,751 adult participants, therapist-guided ICBT was associated with greater improvement compared to self-guided treatment [15]. However, self-guided ICBT indicated comparable efficacy in participants with mild or sub-threshold depression. There are fewer studies that have investigated the importance of therapist support for adolescents and results are inconclusive [14]. To the best of our knowledge, there has not yet been any qualitative research that has explored participant experiences of ICBT without therapist support.

We recently completed a pilot study in which adolescent participants were randomised to guided online BA, self-guided online BA or treatment as usual [16]. The results showed that the online treatments were feasible and preliminarily effective in reducing depressive symptoms. However, when developing new treatments, it is also important to gain knowledge through the experiences of the participants. Through an understanding of the experience of the users, conclusions can be drawn regarding ways in which to further improve treatment [12]. The primary aim of this study was to report and describe the experiences of adolescents receiving online BA treatment, as well as that of their parents in supporting their adolescent through treatment. In addition, the secondary aim of this study was to explore experiences of guided and self-guided BA.

## Methods

### Theoretical framework

Reflexive thematic analysis was used as a flexible method for developing, analysing and interpreting patterns across qualitative data [17, 18]. Reflexive refers to the fact that researchers use their own experience and pre-existing knowledge in the analysis of data.

### Research team

Most interviews were conducted by two female final-year Master’s students in psychology (HW and IU) with basic training in the interview technique. They conducted all but one interview and had no prior contact with the respondents. The final interview was conducted by the first author (RA), a female clinical psychologist who acted as project coordinator and thus had contact with all participating families prior to the interviews. The last author (HE) is a female MD and PhD has extensive experience in qualitative methods and an interest in mental health but had no prior contact with the respondents or prior experience of ICBT. Author SV is a female PhD and trained clinical psychologist. Author JÅ is a male PhD and trained clinical psychologist. Author ES is a female MD and professor in child and adolescent psychiatry. All three latter authors have previous experience of research on ICBT and mental ill-health in adolescents.

### Study design

#### Participant selection

Respondents were recruited from a pilot study on guided or self-guided online BA for mild to moderate depression [16]. Stratified purposive sampling was used to obtain a heterogeneous sample regarding intervention, age, sex and responder status. The aim of this approach was achievement of richness and depth in the data through the exploration of a range of perspectives. After completing the intervention, adolescents and their parents were invited face-to-face or by telephone to participate in the study. Two adolescents declined participation due to lack of time. Informed consent was obtained from all individual participants included in the study. A total of 8 adolescents and nine parents from 11 family units were interviewed (*n* = 17). Five families had participated in self-guided online BA and six in guided online BA. Adolescents were between 13 and 17 years old (mean = 15.2, SD = 1.5), five were male and three were female. All parents interviewed were mothers. Six of 11 adolescents were categorised as treatment responders according to the clinician-rated Clinical Global Impression Scale–Improvement (CGI-I), defined as scores of 1 (very much improved) or 2 (much improved) post-treatment. Respondents with varying degrees of treatment completion (ranging between 25 and 100% module completion) were included [19].

### Data collection

Semi-structured interviews took place between March and October 2020. Separate topic guides were developed for both adolescents and parents (see supplementary table S2). The guides were developed on the basis of previous research and the clinical experience of the authors [12, 14, 20, 21]. The research team discussed the topic guides until consensus was reached. The final topic guides explored (1) treatment expectations, (2) experiences of the BA model, (3) collaboration with the psychologist or participating in treatment without therapist support, (4) parental involvement in treatment, (5) perceived changes following treatment. The guides included both open-ended interview questions asked of everyone and follow-up questions to elicit more comprehensive answers if required. The final versions were pilot-tested in the first two interviews and no changes were deemed necessary. Two interviews were conducted at a clinic for Child and Adolescent Mental Health Services in Stockholm, Sweden. Due to the outbreak of COVID-19, the remaining interviews were conducted online or via telephone. Adolescents and their parents were interviewed separately and there was no one else present during the interview aside from the respondent and the interviewer. During the interviews, participants were not provided any information about the interviewers, such as their personal goals for conducting the research.

Interviews were audio recorded and transcribed verbatim. Field notes were taken during and after the interviews. The interviews lasted between 17 and 62 min (median = 35 min) and were terminated when respondents expressed that they had no more to say. After 17 interviews, the data collected (10 h and 2 min) was considered adequate for richness and complexity as the reflexive thematic analysis framework argues that meaning is generated through interpretation of data and not data saturation [22]. All respondents consented to contact after the interviews for follow-up questions. However, there were no repeat interviews conducted and transcripts were not returned to respondents for comments. The study was authorised by the Swedish Ethical Review Authority.

### The intervention

The online BA interventions (guided and self-guided) were delivered through a secure online platform [16]. Each of the online BA interventions consisted of eight chapters with age-appropriate texts, video clips, fictional characters to relate to and exercises delivered over 10 weeks. The adolescents worked with an app to assist with assignments in between chapters. An automated reminder was sent to participants who had been inactive for 2 days. A parallel 10-week parental course that was either guided or self-guided depending on group allocation was included in the treatment. Adolescents and their parents received separate login details for the platform. In two-parent families, both parents were encouraged to participate. The main purpose of the parental programme was to help parents to support their adolescents in engaging in treatment. The parental course also included positive parenting skills, intended to strengthen the relationship between parents and their children. An overview of the treatment chapters is presented in supplementary Table S3.

In the guided online BA, participants had regular contact with a clinical psychologist via written messages on the platform and occasional phone calls. The psychologist logged into the online platform at least every other day during workdays to provide guidance, answer questions, clarify the treatment rationale and prompt inactive participants to login again. Asynchronous communication took place via text message or comments on exercises. The psychologist spent an average of 23 min (SD = 6) on each treatment chapter for each family, including sending messages on the platform and occasional telephone calls. In contrast, for the self-guided online BA adolescents and their parents did not have access to any support. However, to ensure patient safety the study team monitored for symptoms of suicidal tendencies for all participants during treatment.

### Data analysis

The analysis consisted of six phases as described by Braun and Clarke [17]: (1) becoming familiar with the data; (2) code generation; (3) theme construction; (4) review of potential themes; (5) definition and naming of themes; and (6) report production. The analysis is not a linear process and the researchers may oscillate between phases as required and as the analysis develops. In the first phase, the transcribed and deidentified interviews were read multiple times in order to become familiar with the data. In the second phase, interviews were coded for interesting features related to the research questions, using an iterative inductive process as there was no pre-existing coding frame used. The first interviews were coded in parallel by IU, HW, RA, SV and HE until consensus was reached, followed by coding of the remaining interviews by IU, HW and RA in the same manner. Reflexive discussions were ongoing and understanding of the data set was debated in the whole research group. In the third phase, the data were used for the generation of themes and sub-themes reflecting the relationship between codes. The analytical process involved moving between data, coding and identifying where themes could be created. In phase four, the individual transcripts and the entire data set were reviewed to confirm that the themes generated reflected the data set. The themes were then defined and the whole research team discussed an appropriate approach to naming the themes in order to help the reader understand the data. Lastly, the results were drafted with the intention of presenting a concise narrative both within and across themes. Data were analysed using MAXQDA 2020 [23]. In addition, the COREQ standard for reporting qualitative data was used when drafting the manuscript [24] (see supplementary table S1).

## Results

**Fig. 1:**
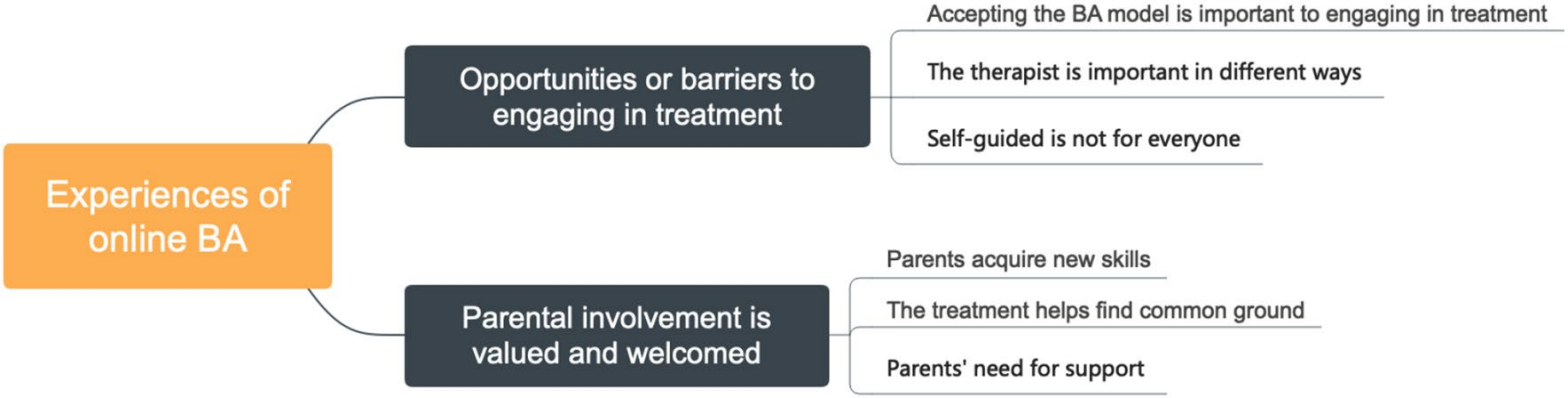
Thematic map of themes and sub-themes

Two overarching themes were identified in the analysis. The first theme explored the aspects that respondents experienced as either facilitating or hindering to engaging in the treatment. The second theme investigates the experience of parental involvement in the treatment. Quotes are added to illustrate each sub-theme and are attributed to individual respondents (A = adolescent, P = parent). An overview of themes and sub-themes is presented in Fig. 1.

### Opportunities or barriers to engaging in treatment

The first theme encompasses experiences that facilitated or hampered treatment engagement.

#### Accepting the BA model is important to engaging in treatment

Respondents in both guided and self-guided groups accepted the BA model of depression to varying degrees. Those who accepted the rational presented in BA, described how inactivity or lack of positive reinforcers contributed to their depression.

*“When you’ve been depressed, maybe haven’t been able to do many of the things that you like doing ... like playing hockey in my case ... and you might stay away from friends, and then eventually you don’t talk as much anymore… you can't cope anymore ... and um... there was a lot of stuff that I recognised in myself....” (A4, guided)*

There were several elements of BA that were experienced as helpful by both adolescents and their parents, such as understanding the association between activities and mood, learn problem-solving skills and how to identify and break vicious cycles of depression, as well as increasing adaptive activites.

*“Once she started this treatment, she became incredibly active and did things. /…/ Every time I came home from work, the kitchen bench was clean. She felt that she was succeeding. And without me having to say anything. /…/ This meant she made it out of that very dark space where she only wanted to stay in bed”.* (P9, self-guided)

Respondents who could relate to the BA model also experienced several positive treatment outcomes for example, reduced depressive symptoms such as less anhedonia and increased positive feelings such as looking forward to things again and setting goals for the future. They also described feelings of increased engagement in important activities, feeling closer to friends and attending school more often.

*“I’ve been able to truly laugh again. I go out with my friends a lot more. I have fun with them; I have fun with my little brother. I wasn’t before”.* (A6, guided)

*”Even though I didn’t register things in the app as often as I wanted to, I still did more things than I usually would. I've been a lot more active with my family and with other things that I'm supposed to do.” (A5, self-guided)*

In contrast, other respondents in both groups found it hard to relate to the BA model and felt that it lacked important components, such as strategies for addressing negative thinking or how to regulate difficult emotions.

*“What am I supposed to do if I’m really sad? It was pretty much ‘talk to someone or listen to music’. So, I tried but I was struggling so much [with depressed feelings that] it just didn’t work for me”.* (A7, guided)

Another reason for low engagement was that some felt that they were already doing everything that mattered to them and as a result could not see the point of engaging in new activities as suggested by the treatment model.

*“I don’t think that doing scheduled activities was that helpful since I'm already doing things I like… it was a bit pointless”.* (A1, self-guided)

Parents also reported difficulties in identifying with the BA model. One reason was not recognising their own adolescent child in the severity of depression illustrated by different examples in the chapters in the treatment.

"Sometimes it felt like [my child's] situation is not as bad as it is supposed to be in this treatment... I didn't always recognize my child in the problems illustrated in the chapters." (P4, self-guided)

Some respondents felt that external circumstances affected their engagement for example, high demands at school, increased workload or extra burden due to illnesses or other negative life events.

*“Um, I don’t really know. It kind of stressed me out a bit, but yeah I kind of understand the purpose of this and that you should do activities and stuff and I understood why that is good. But for me, I ended up being so stressed out about school… so this whole planning everything and whatever, just stressed me out more… so I ended up not doing much at all”. (A2, self-guided)*

Other respondents described how working with the treatment became part of their daily routine and made it easier to remain engaged.

*“At first, it was a bit difficult to log in and then have to do it every week, but once you did it then it was like a routine that you had to do anyway, so that’s just how I did it and it was easier to do it.” (A3, guided)*

#### The therapist is important in many ways

In the guided BA group, access to a therapist was experienced as important for engagement by both adolescents and parents. The therapist assisted participants in making the content of the treatment understandable and relevant to the unique situation of each respondent.

*“If there was something I was wondering about, I could just ask her [the therapist] and she would answer right away... and kind of give me good tips and stuff... so I feel like I’ve been able to express everything that I’ve been feeling and ask for help when I needed it.” (A4, guided)*

*“I got feedback from the psychologist really quickly, things like 'good idea’ or ‘oh yes, if you think that about this parenting trap, then you could maybe try doing this’, so the times I’ve asked for help I’ve been given great feedback”. (P3, guided)*

Second, both adolescents and parents described how the therapist was important for maintaining motivation and engagement in the treatment. For example, by reminding the adolescent to log into the platform and encouraging their efforts with assignments and changing behaviour.

*"For me, it was a very pleasant surprise that you also could have such communication via the internet. That you could ask questions or that she [the therapist] noticed if there was something that you had been thinking about. And I got great feedback. On that front, I've been very satisfied. It was beyond expectation. It was a privilege.” (P6, guided)*

#### Self-guided is not for everyone

In the self-guided BA group, some adolescents and their parents reported benefits from working without therapist support. Several reasons were given, including that they did not need a therapist, that it was nice to be able to take in the material at their own pace or that they did not like talking to people. Some explained that they had previously had negative experiences with face-to-face therapy and that the online format helped them to focus on change, rather than ruminating or dwelling on the past.

*“I think that if we had ended up in the group with psychologist support, it could’ve stopped his progression. Because when he went to see the school psychologist, he felt like he’d just delved into his past without getting any further”.* (P8, self-guided)

*“It [self-guided] worked well. (…) I think this has worked best for me (…) I feel like I got what I needed”.* (A6, self-guided)

On the other hand, some respondents in the self-guided BA had difficulty following the treatment plan and felt left on their own. Furthermore, they expressed that they thought a therapist would have helped them overcome challenges such as difficulty recognising themselves in the fictional characters in the treatment. This lack of support reduced motivation and commitment to treatment.

*“When it's on the internet, it's saying something quite general about someone's problems... and maybe you don’t always recognise yourself in it... But, like if you have a psychologist that you talk to, they would know everything about your problem and like why...or yeah, all about the patient’s depression...”* (A2, self-guided)

Other parents were initially sceptical about self-guided BA, thinking it would be too demanding for both their child and themselves. This changed after the parent saw the adolescent working independently in treatment and at their own pace. However, all parents including those who considered self-guided to be the best option for their child, wanted therapist support for themselves.

*“I would’ve liked to have had someone to use as a sounding board. Instead, I had to use my partner. That’s not a bad thing. But I think I needed it [a therapist]. After all, he’s part of all of this. You end up not being able to see the wood for the trees, so someone on the outside that I could talk to would’ve been good”.* (P8, self-guided)

### Parental involvement is valued and welcomed

All parents expressed the fact that they valued their participation in the treatment. However, adolescents’ experiences of parental involvement varied from barely noticing the participation of their parents to greatly welcoming their involvement. None of the adolescents experienced parental involvement as negative or unwanted.

#### Parents acquire new skills

Parents reported experiencing several positive aspects of being involved in the treatment. They stated that they gained tools for use in everyday life such as nagging less, encouraging more, and seeing things from the perspective of their child. They also learnt how to manage their own frustration, push their child in a helpful way, make reasonable demands and adjust their level of ambition.

*“When I lower the bar, then she [the adolescent] will often raise it a little herself, as she’s ambitious. She'll then find the self-motivation in another way”.* (P9, self-guided)

Parents also reported that being involved in treatment strengthened the parent–child relationship and brought them closer together. Both adolescents and parents described how they could now talk to each other more easily and cooperate when dealing with difficult situations, such as not going to school due to depression. The adolescents also reported that their parents asked how they were feeling more often, were more accepting and that they pushed and supported them in a more empathetic way.

*“He [the dad] used to really like, push me to go and work out… which made it difficult. But now he says that I should go at my own pace and that I shouldn’t feel that like there’s any pressure from him… they [the parents] have also changed and that’s helped me.”* (A4, guided)

Other parents reported feeling frustration over parts of the parental course that they already knew and initially feel patronised. This frustration later turned into feeling validated.

*"At the beginning, it was more like: ‘don’t they think I'm smarter than this?’. /.../ But then, when you get a little further into the treatment, it became more like, ‘Aha, okay, that’s nice’... /.../ I could relax and I know that I’m doing my best for him [the adolescent]. I’m doing all I can. (P8, self-guided).*

An additional benefit of participation in the parental course was that some parents came to the realisation that they had mental health issues of their own:

*“The course provided a clear description of what depression is and how you can overcome it. You actually need to take small steps forward (…). It was great because it made my husband realise that he is [also] depressed” ... And then we thought that’we have to do something about the situation. Because otherwise we'll all be depressed and no one will be able to do anything about it. It will affect the whole family... We decided to do this properly. That we’ll do it together-as a family (P5, guided).*

#### The treatment helps find common ground

In most families, only one parent actively participated. However, the other parent was involved in different ways. Most often, the active parent conveyed course material verbally to the other parent. Some found passing on insights to the other parent to be easy owed to the relatively readily understandable contents.

*“This treatment uses simple language–it’s the kind you can relate to, so it’s not psychology that’s hard to understand. It’s common sense, about how we treat each other and about behaviour. So I don’t think it’s been difficult to pass that on to the other parent.” (P6, guided).*

Parents who participated together experienced that active participation from both parents was crucial. Their experience was that they were more receptive to information when it came from an external source. Parents described feeling supported by one another and finding it easier to prioritise treatment when working together.

*“We could do things together. We could discuss things together. 'What do we need to do here? This exercise didn’t have the results we wanted, why is that?' Or, if we had not done a certain task, we could discuss why”.* (P5, guided)

In addition, parental participation in the treatment was valued by both parents and adolescents as it brought a sense of shared responsibility for the recovery of the adolescent from their depression and facilitated reaching a common understanding of the struggles the adolescent was working through.

*“There were a few times where she [the adolescent] has been able to come and ask things like, 'What have you done' and there would be a little bit of the feeling that the two of us [the parents] have worked together for her well-being...” (P3, guided)*

*"I noticed that she [the mum] felt better and that she could handle my condition better. Because before the treatment, we didn’t know what was wrong with me. It often ended up with us just yelling at each other and arguing." (A6, self-guided)*

In contrast, other adolescents reported that they did not reflect much about the work of their parents with the treatment.

*“I don’t usually notice my parents that much, to be completely honest.” (A1, self-guided)*

#### Parents’ need for support

Parents felt that contact with a therapist meant they could share the burden of having a child struggling with their mental health. Several parents reported that it felt safe and professional for their child to have contact with a therapist. It was a relief to be able to share the responsibility of their depressed teenager with someone.

*“OK, it feels wrong to say this, but it’s been nice that I’ve not needed to take all the responsibility, I’ve been able to take a step back”.* (P3, guided)

Moreover, some parents experienced a need for a therapist to help them cooperate and reach a common understanding of the challenges faced by the child.

*“His father and I have a completely different interpretation of what constitutes reasonable screen time. (…) It would’ve been optimal if both his father and I could have cooperated more”.* (P1, self-guided)

## Discussion

This study explored adolescents' and their parents’ experiences with online BA for depression with and without therapist support. The analysis showed that respondents who could identify with the BA model of depression–regardless of whether online BA was conducted with or without therapist support–were more engaged in treatment. Correspondingly, respondents who thought that they were already doing what mattered to them struggled to engage in treatment. Furthermore, although BA is described as straightforward and easy to understand [3], respondents in this study show that BA can also be seen as too simplistic and unable to capture the complexity of their problems. The importance of identification has been reported in a previous systematic review of studies on adult experiences of ICBT [25]. The review concluded that the more personalised the content of the treatment, the greater its effect. Identification may be especially important in internet-delivered treatment. In face-to-face treatment, session content is co-created on a continual basis using both verbal and non-verbal communication; the therapist provides expert knowledge, while the client provides experiences and observations from their life. In contrast, ICBT materials are standardised and more generalised, leaving it up to the user to apply the model to their own situation and to choose the aspects that they find useful. Therapist support in ICBT can help tailor the treatment [21] and thus, self-guided formats might suffer more in this respect compared to guided formats.

In this study, life stressors such as negative life events, academic pressure and increased parental workload were found to be barriers to engaging in treatment. The challenge of making room for treatment in everyday life has been recognised in previous research on BA for adolescents, where obstacles included anxiety, fatigue, school requirements and lack of motivation [5]. While ICBT is deemed flexible, it lacks the framework that arises naturally in a face-to-face treatment with a therapist in regular planned sessions [24]. This places great demands on the patient to plan and prioritise the treatment themselves. Furthermore, prioritising treatment can be even more difficult in depression where lack of motivation is a core symptom. This poses a particular challenge in self-guided formats in which there is no therapist present to react to late assignments or lack of engagement.

Our results showed that parents valued being involved in the treatment as they learned how to both support their depressed adolescents and strengthen their parent–child relationship. Previous research suggests that parental involvement might improve long-term clinical outcomes in treatment for adolescent depression [26, 27] and Grist et al. found that internet-based interventions were more effective if parents were involved [28]. Given that lack of motivation is often a symptom of depression, parental involvement may be even more important in online BA, particularly in self-guided formats compared to other ICBT interventions. Furthermore, parents are an important part of the adolescent context and are essential in facilitating activation. Although this study is not able to draw any conclusions on the clinical effect of parental involvement, it is important to highlight that involving parents in the treatment of adolescent depression is experienced as helpful and makes parents feel more confident in how to support their adolescent [21].

Although therapist support has previously been shown to be both appreciated [20, 21] and associated with better clinical outcomes [29], the benefits of self-guided ICBT are significant in terms of scalability. A recent meta-analysis also found that self-guided ICBT may be an adequate treatment for those with sub-threshold and milder problems [30] and consequently self-guided online BA might suffice for adolescents with milder depression. We found that respondents in guided online BA appreciated the practical and emotional support and associated therapist support with increased motivation. Parents also highlighted the emotional support and felt a sense of relief in being able to share the responsibility for the well-being of their child. In self-guided BA, some respondents found the lack of access to a therapist to be lonely and disengaging, while others reported feeling empowered and in control. Importantly, adolescents who expressed a preference for working without therapist support might actually be comparing online treatment to face-to-face therapy, as they had no previous experience of therapist-guided online treatment. In summary, although no direct comparison can be made between the guided and self-guided groups, therapist support seems to be important for engagement and for emotional support for parents. This new knowledge may prove useful to help improve understanding of patient preferences for guided versus self-guided BA.

### Strengths and limitations

This study has several strengths. The first is that both parents and adolescents participated in this study and provided two perspectives on experiences of online BA for depression. Also, the perspectives of the self-guided group and those of the group with therapist support were included. The second is that experiences of both treatment responders and non-responders were included, which is useful when adapting or expanding an intervention to fit more adolescents with depression. One limitation is that the inclusion of several participant groups may have made analysing data more difficult and presentation of results less clear. Another limitation is that this study included only adolescents with mild to moderate depression. Hence, differing results may emerge from a population with more severe depression. Also, the sample size for each subgroup was small and it might limit the transferability of the results.

Further to this, qualitative research is sensitive to interviewer bias. The two main interviewers (HW and IU) were not involved in treatment development nor in working as therapists in the feasibility trial. Nevertheless, they were Master’s students of clinical psychology with an interest in CBT and certain preconceptions may have impacted the data. Given the qualitative design, different findings may emerge from studies with participants with different characteristics,  for example, in terms of the clinical setting and patient group [31].

### Future studies

As this is the first qualitative study exploring experiences of adolescents and parents in guided or self-guided online BA, there is a considerable need for further research. The findings in this study will benefit from exploration in other  samples and in other contexts. If self-guided BA is found to be an effective treatment, one challenge for future studies will be identification of patient groups for whom it is best suited. Another future direction is the possibility of investigating a stepped care approach, exploring when therapist-guidance or face-to-face treatment would be most beneficial. The study found several benefits of parental involvement, which raises interesting questions for future studies. For example, ‘Does parental involvement contribute to larger treatment effects? Or does it impact parental well-being If so, are there any health economic implications?'

## Conclusion

Adolescents find online BA to be an engaging and helpful treatment, but some adolescents struggle to identify with the BA model. In addition, the adolescents welcomed parental involvement in their treatment and participation was also important to the parents. The results highlight several advantages of involving parents in treatment, such as skill acquisition and an improved parent–child relationship. Finally, therapist support is experienced as important for engaging and maintaining motivation throughout the treatment, with some respondents in self-guided BA expressing an explicit wish for therapist support. Parents in particular emphasised the need for therapist contact for emotional support, as well as to facilitate cooperation between parents. Our findings indicate that guidance from a therapist is a valued part of the experience for those in internet-delivered BA treatment.

## Supplementary Information

Below is the link to the electronic supplementary material.

Supplementary file1 (DOCX 30 KB)

## Data Availability

No data are available. The data are pseudonymised according to national (Swedish) and European Union legislation, and cannot be anonymised and published in an open repository. Participants in the trial have not consented for their data to be shared with other researchers for research purposes.
