# Supplementary material for: “Therapy without a therapist?” The experiences of adolescents and their parents of online behavioural activation for depression with and without therapist support
Source: Eur Child Adolesc Psychiatry. 2023 Jan 17:1–10. Online ahead of print. doi: 10.1007/s00787-023-02142-7 (PMC9844942; doi:10.1007/s00787-023-02142-7)
Supplement: Supplementary file 1 [file 787_2023_2142_MOESM1_ESM.docx]

# “Therapy without a therapist?” Adolescents’ and their parents’ experiences of online behavioural activation for depression with and without therapist support

Journal name: European Child & Adolescent Psychiatry

**Rebecca Grudin**^**1,***^**, Sarah Vigerland**^**1**^**, Johan Ahlen**^**2,3**^**,** **Hanna Widström**^**4**^**, Irma Unger**^**5**^**, Eva Serlachius**^**6,7**^**, Hedvig Engberg**^**8,9**^

^1^Centre for Psychiatry Research, Department of Clinical Neuroscience, Karolinska Institutet, & Stockholm Health Care Services, Region Stockholm, CAP Research Centre, Gävlegatan 22, SE-113 30 Stockholm, Sweden;  ^2^The Centre for Epidemiology and Community Medicine, Region Stockholm, Box 45436, SE-104 31 Stockholm, Sweden; ^3^Department of Global Public Health, Karolinska Institutet, SE-171 77 Stockholm, Sweden; ^4^Moment Psychology, Drottninggatan 99, SE-113 60 Stockholm, Sweden; ^5^Wemind Psychiatry, Rehnsgatan 20, SE-113 57, Stockholm; ^6^Department of Clinical Neuroscience, Karolinska Institutet, SE-171 77 Stockholm, Swede; ^7^Department of Clinical Sciences, Faculty of Medicine, Lund University, Baravägen 1, SE-222 40 Lund, Sweden; ^8^Department of Women’s and Children’s Health, Karolinska Institutet, Stockholm, Sweden;  ^9^Department of Obstetrics and Gynaecology, Karolinska University Hospital, Stockholm, Sweden 

*Corresponding author: [rebecca.grudin@ki.se](mailto:rebecca.grudin@ki.se), ORCID: 0000-0002-0284-0893

**Supplementary table 1**: Consolidated criteria for reporting qualitative studies (COREQ): 32-item checklist

Developed from:

Tong A, Sainsbury P, Craig J. Consolidated criteria for reporting qualitative research (COREQ): a 32-item checklist for interviews and focus groups. *International Journal for Quality in Health Care*. 2007. Volume 19, Number 6: pp. 349 – 357

| **No.  Item** | **Guide questions/description** | **Reported on Page #** |
| --- | --- | --- |
| **Domain 1: Research team and reﬂexivity** |  |  |
| *Personal Characteristics* |  |  |
| 1. Interviewer/facilitator | Which author/s conducted the interview or focus group? | Methods, p. 4 |
| 2. Credentials | What were the researcher’s credentials? E.g. PhD, MD | Methods, p. 4. |
| 3. Occupation | What was their occupation at the time of the study? | Methods, p. 4 |
| 4. Gender | Was the researcher male or female? | Methods, p. 4 |
| 5. Experience and training | What experience or training did the researchers have? | Methods, p. 4 |
| *Relationship with participants* |  |  |
| 6. Relationship established | Was a relationship established prior to study commencement? | Methods, p. 4 |
| 7. Participant knowledge of the interviewer | What did the participants know about the researcher? E.g., personal goals, reasons for doing the research | Methods, p. 5 |
| 8. Interviewer characteristics | What characteristics were reported about the inter viewer/facilitator? E.g., bias, assumptions, reasons and interests in the research topic | Discussion/Strengths and limitations, p. 17 |
| **Domain 2: study design** |  |  |
| *Theoretical framework* |  |  |
| 9. Methodological orientation and Theory | What methodological orientation was stated to underpin the study? E.g., grounded theory, discourse analysis, ethnography, phenomenology, content analysis | Methods, p. 4 |
| *Participant selection* |  |  |
| 10. Sampling | How were participants selected? E.g., purposive, convenience, consecutive, snowball | Methods, p. 4-5 |
| 11. Method of approach | How were participants approached? E.g., face-to-face, telephone, mail, email | Methods, p. 5 |
| 12. Sample size | How many participants were in the study? | Methods, p. 5 |
| 13. Non-participation | How many people refused to participate or dropped out? Reasons? | Methods, p. 5 |
| *Setting* |  |  |
| 14. Setting of data collection | Where was the data collected? E.g., home, clinic, workplace | Methods, p. 5 |
| 15. Presence of non-participants | Was anyone else present besides the participants and researchers? | Methods, p. 5 |
| 16. Description of sample | What are the important characteristics of the sample? E.g., demographic data, date | Methods, p. 5 |
| *Data collection* |  |  |
| 17. Interview guide | Were questions, prompts, guides provided by the authors? Was it pilot tested? | Methods, p. 5 |
| 18. Repeat interviews | Were repeat inter views carried out? If yes, how many? | Methods, p. 6 |
| 19. Audio/visual recording | Did the research use audio or visual recording to collect the data? | Methods, p. 5 |
| 20. Field notes | Were ﬁeld notes made during and/or after the interview or focus group? | Methods, p. 5 |
| 21. Duration | What was the duration of the interviews or focus group? | Methods, p. 5 |
| 22. Data saturation | Was data saturation discussed? | N/A, since the concept of saturation is not consistent with the assumptions of reflexive thematic analysis. This is commented on p 6. |
| 23. Transcripts returned | Were transcripts returned to participants for comment and/or correction? | Methods, p. |
| **Domain 3: analysis and ﬁndings** |  |  |
| *Data analysis* |  |  |
| 24. Number of data coders | How many data coders coded the data? | Methods, p. 7 |
| 25. Description of the coding tree | Did authors provide a description of the coding tree? | Methods, p. 7 |
| 26. Derivation of themes | Were themes identiﬁed in advance or derived from the data? | Methods, p. 7 |
| 27. Software | What software, if applicable, was used to manage the data? | Methods, p. 7 |
| 28. Participant checking | Did participants provide feedback on the ﬁndings? | Methods, p. 6 |
| *Reporting* |  |  |
| 29. Quotations presented | Were participant quotations presented to illustrate the themes/ﬁndings? Was each quotation identiﬁed? E.g. participant number | Results, pp. 7–15 |
| 30. Data and ﬁndings consistent | Was there consistency between the data presented and the ﬁndings? | Results, pp. 7–15 |
| 31. Clarity of major themes | Were major themes clearly presented in the ﬁndings? | Results, pp. 7–15 |
| 32. Clarity of minor themes | Is there a description of diverse cases or discussion of minor themes? | Results, pp. 7–15 |

## **Supplementary table 2:** Overview of the treatment chapters in online BA

| **Chapter** | **Adolescent** | **Parent** |
| --- | --- | --- |
| 1 | Introduction to online BA. Psychoeducation on depression. The rationale for BA. *Homework*: activity monitoring. | Introduction to online BA. Psychoeducation on depression. The rationale for BA. Learn about common parent pitfalls. *Homework*: reflect on personal parental behaviours when the adolescent shows depressive behaviour. Discuss with the adolescent how to cooperate in treatment. |
| 2 | Values assessment. Set treatment goals. *Homework*: activity scheduling. | Facilitate and encourage values-based activation; communication skills I: validate your child’s feelings. *Homework:* practice validating other’s and your child’s emotions, encourage values-based activation. |
| 3 | Continued values-based activation. Psychoeducation on sleep. *Homework*: activity scheduling and sleep hygiene. | Spending positive time with your adolescent. *Homework*: suggest positive time with your adolescent |
| 4 | Continued values-based activation. Identify and overcome barriers to activation through identifying and overcoming avoidance. *Homework*: activity scheduling, sleep hygiene, and practice overcoming avoidance. | Communication Skills II: How to avoid and manage conflicts. *Homework*: practice conflict management. |
| 5 | Continued values-based activation. Overcome barriers to activation through shifting focus to the present situation. *Homework*: activity scheduling, sleep hygiene, and practice shifting focus. | Take care of yourself as a parent supporting a child with depression. *Homework*: take care of yourself. |
| 6 | Continued values-based activation. Problem-solving. *Homework*: activity scheduling, sleep hygiene, and practice problem-solving. | Collaborative problem-solving. *Homework*: practice collaborative problem-solving |
| 7 | Putting it all together. *Homework*: activity scheduling. | Putting it all together. *Homework*: choose two tasks from previously introduced skills. |
| 8 | Treatment summary. Relapse prevention. Evaluate the treatment. | Course summary. Relapse prevention. Evaluate treatment. |

**Supplementary table 3:** Topic guides for adolescents and their parents 

*The questions below were adapted when interviewing the parents.*

| **Area of interest** | **Fixed questions** | **Suggestive questions** |
| --- | --- | --- |
| Experience of treatment | Would you like to share your experience of the treatment? Tell me more. |  |
| Expectations before treatment | What expectations did you have before starting treatment? | What did you hope for? Thoughts about internet-delivered versus face-to-face treatment? Were your expectations met? |
| Recognition of treatment rationale | What do you think of the treatment material about depression? | Could you identify with how depression was described in the treatment? How come? What could you relate to? What could you not relate to? |
| Treatment content | How did you experience the content in the treatment? | Could you describe what was helpful to you in the treatment? What was less helpful to you? |
| Parents participating | Please tell me what it was like having your parent(s) participate in the programme? | Did you cooperate? If so, in what way? Did you notice any differences in your parent’s attitudes or behaviours? |
| Collaboration with the psychologist | Did you have contact with a therapist during treatment? *If yes:* What did you appreciate? What did you miss in your contact? *If no*: What are your thoughts about that? |  |
| Change due to treatment | Has anything changed in your life, compared to before the treatment? If yes, how so? | Symptoms? Perceptions of symptoms? In school? With friends or family? In your spare time? Self-esteem? Life quality? |
